# Promising Long-Term Residential Care Policy Guidance for Staff to Support Resident Quality of Life

**DOI:** 10.1093/geroni/igab046.1427

**Published:** 2021-12-17

**Authors:** Mary Jean Hande

**Affiliations:** Mount Saint Vincent University, Halifax, Nova Scotia, Canada

## Abstract

This paper reviews 63 policy documents in four Canadian jurisdictions that guide long term residential care staff on how to enhance 11 resident quality of life in Canada. We found guidance in each jurisdiction that provide clear language to support staff discretion and flexibility to navigate regulatory tensions and enhance resident quality of life. Newer policies tend to reflect more interpretive approaches to staff flexibility and broader quality of life concepts. We argue that if interpreted through a resident quality of life lens and with the right structural supports, these promising texts offer important counters to the rigidity of long term residential care policy landscape and can be leveraged to effectively broaden and enhance quality of life for residents in long term residential care.

